# Transposon-sequencing across multiple *Mycobacterium abscessus* isolates reveals significant functional genomic diversity among strains

**DOI:** 10.1128/mbio.03376-24

**Published:** 2024-12-31

**Authors:** Chidiebere Akusobi, Sanjeevani Choudhery, Bouchra S. Benghomari, Ian D. Wolf, Shreya Singhvi, Thomas R. Ioerger, Eric J. Rubin

**Affiliations:** 1 Department of Immunology and Infectious Diseases, Harvard T.H. Chan School of Public Health, Boston, Massachusetts, USA; 2 Department of Computer Science and Engineering, Texas A&M University, College Station, Texas, USA; 3 Department of Biology, Northeastern University, Boston, Massachusetts, USA; 4 Department of Molecular and Cell Biology, University of California, Berkeley, Berkeley, California, USA; Washington University in St. Louis School of Medicine, St. Louis, Missouri, USA

**Keywords:** *Mycobacterium abscessus*, genetic diversity, transposon-sequencing, essential genes, functional diversity

## Abstract

*Mycobacterium abscessus (Mab*) is a clinically significant pathogen and a highly genetically diverse species due to its large accessory genome. The functional consequence of this diversity remains unknown mainly because, to date, functional genomic studies in *Mab* have been primarily performed on reference strains. Given the growing public health threat of *Mab* infections, understanding the functional genomic differences among *Mab* clinical isolates can provide more insight into how its genetic diversity influences gene essentiality, clinically relevant phenotypes, and importantly, potential drug targets. To determine the functional genomic diversity among *Mab* strains, we conducted transposon-sequencing (TnSeq) on 21 genetically diverse clinical isolates, including 15 *M*. *abscessus* subsp. *abscessus* isolates and 6 *M*. *abscessus* subsp. *massiliense* isolates, cataloging all the essential and non-essential genes in each strain. Pan-genome analysis revealed a core set of 3,845 genes and a large accessory genome of 11,507. We identified 259 core essential genes across the 21 clinical isolates and 425 differentially required genes, representing ~10% of the *Mab* core genome. We also identified genes whose requirements were subspecies, lineage, and isolate-specific. Finally, by correlating TnSeq profiles, we identified 19 previously uncharacterized genetic networks in *Mab*. Altogether, we find that *Mab* clinical isolates are not only genetically diverse but functionally diverse as well.

**IMPORTANCE:**

This study investigates the genetic diversity of *Mycobacterium abscessus* (*Mab*), a bacteria known for causing difficult-to-treat infections. Researchers performed transposon-sequencing (TnSeq) on 21 different clinical isolates of *Mab* to identify essential and non-essential genes in each strain. Through this analysis, they identified core genes required for growth across all strains. Interestingly, they also identified genes whose requirement for growth or “essentiality” were subspecies, lineage, and isolate-specific. This study reveals that *Mab’*s genetic diversity translates into significant functional differences among clinical isolates. Insights from this paper lay essential groundwork for future studies exploring the biological and clinical implications of genetic diversity in *Mab* clinical isolates. Understanding this diversity could guide targeted therapies and offer new insights into managing infections caused by *Mab*, a growing public health concern.

## INTRODUCTION

*Mycobacterium abscessus (Mab*) has emerged in recent years as a growing threat to public health due to the rise of *Mab* infections worldwide (1). *Mab* causes a wide range of infections including 80% of pulmonary infections from rapidly growing mycobacteria (2, 3). Treating *Mab* infections is difficult due to *Mab’s* high level of intrinsic resistance to many antibiotics, including most of the cornerstones of tuberculosis treatment (4–7). Importantly, treating *Mab* requires identifying the subspecies of the clinical isolate, which influences antibiotic treatment based on the presence of *erm*, a gene that confers inducible macrolide resistance (8, 9).

*Mab* is divided into three subspecies: *abscessus, bolletii,* and *massiliense* (10, 11). Notably, most *massiliense* isolates lack an active *erm* gene (8, 9). Whole-genome sequencing of *Mab* clinical isolates revealed that *Mab* has a large, open pan-genome, with isolates sharing ~75% of their genome in what is known as the “core genome” (12, 13). The remaining 25% of gene content is in the “accessory genome” defined as genes present in some clinical isolates but not all. Most accessory genome content comprises plasmids, prophages, and genomic islands acquired by horizontal gene transfer (HGT) (14). Unlike *Mycobacterium tuberculosis* (*Mtb*)*,* which evolves clonally likely due to its facultative intracellular lifestyle (15), the primary driver of *Mab* evolution is recombination and HGT (12, 16, 17). As a result, compared to TB, *Mab* clinical isolates have larger accessory genomes, which are significant contributors to the genetic diversity present in this species.

While *Mab* clinical isolates have large accessory genomes, the functional consequence of this diversity remains unknown. Previous studies have shown that accessory gene content impacts genetic networks, generating new phenotypes, gene dependencies, and redundancies within strains (18–20). Variation in accessory genomes likely generates functional genomic and phenotypic differences between isolates. The functional differences between isolates can be understood through genetic screens using CRISPRi and transposon-sequencing (TnSeq) libraries. TnSeq and the subsequent identification of essential genes have typically been conducted in lab-adapted reference strains. However, in recent years, groups have performed TnSeq on clinical isolates of pathogens, including *Salmonella enterica, Streptococcus pneumoniae, Staphylococcus aureus,* and *M. tuberculosis*. These studies identified genes that were differentially essential among clinical isolates and that these differences influenced clinically relevant phenotypes (19, 21–24).

For *Mab*, the majority of TnSeq studies published to date have used the lab-adapted reference strain, ATCC 19977 (25–27). One TnSeq study was conducted on a *massiliense* clinical isolate; however, the transposon library was not dense enough to quantify genetic requirements (28). Currently, there are no published studies investigating the functional diversity of *Mab* clinical isolates. To accomplish this, we conducted TnSeq on 21 *Mab* strains, generating triplicate libraries for 15 *abscessus* isolates and six single libraries of the *massiliense* isolates. Across the 21 strains, we identified 425 genes, representing ~15% of the *Mab* core genome, as differentially required among isolates. We also identified genes whose differences in genetic requirements were subspecies, lineage, and isolate-specific. Finally, we identified pairs of genes whose genetic requirements were correlated, revealing previously undescribed genetic networks in *Mab*. Overall, we find that the genetic diversity in *Mab* clinical isolates is matched with significant functional genomic diversity as well.

## RESULTS

### Characterization of *Mab* clinical isolates

**Fig 1:**
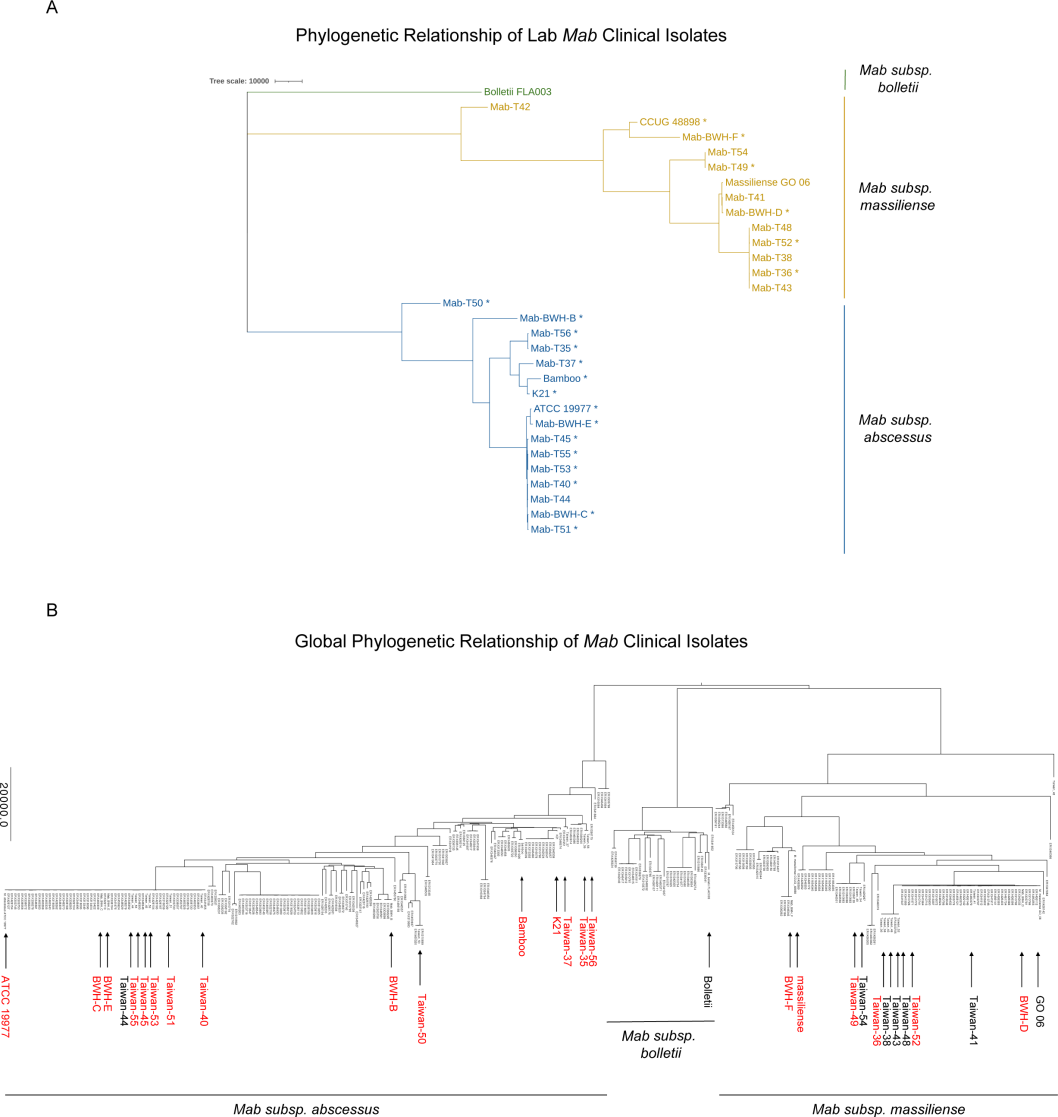
Phylogenetic relationship of *Mab* clinical isolates. (**A**) Phylogenetic tree of 25 Mab clinical isolates and five reference strains. The tree is generated based on genome-wide SNPs, using Gubbins to filter out genomic regions potentially affected by recombination. Strains with transposon-mutant libraries are marked with an “*.” (**B**) Global phylogenetic tree of *Mab* clinical isolates using genomes of 307 clinical isolates from reference (11). Strains in the lab are labeled with an arrow. Red arrows indicate strains that have transposon-mutant libraries.

Our lab obtained 25 *Mab* clinical isolates previously collected from patients in Taiwan and locally at Brigham and Women’s Hospital (BWH) (Table S1, https://orca2.tamu.edu/Mab_TnSeq/). The clinical isolates represented a convenient sample of isolates to functionally characterize, given that they were isolated from multiple sites of infection, included both smooth and rough colony-morphology variants, and comprised both *abscessus* and *massiliense* subspecies. We passaged the clinical isolates only once to decrease the selection of mutants under *in vitro* growth conditions. We then determined the genetic diversity of our clinical isolates through whole-genome sequencing (WGS), followed by *de novo* assembly using ABySS (29) and annotation of the genomes using RAST (see Materials and Methods). The average number of nucleotides in the assemblies was 5,103,719 bp. Summary statistics of the WGS data are presented in Table S2 (https://orca2.tamu.edu/Mab_TnSeq/). A phylogeny of the 25 clinical isolates and five reference strains is shown in Fig. 1A. The tree was generated using genome-wide single-nucleotide polymorphism (SNP) data while excluding SNPs from regions that showed evidence of a high degree of recombination, as determined by Gubbins (30). To put these isolates into a broader genomic context, they were incorporated into a global phylogeny constructed from 307 *Mab* isolates from Bryant et al. (11) using TNT (31). The resulting tree recapitulated the three subspecies of *Mab*, with our clinical isolates distributed across the entire tree (Fig. 1B). About 60% of our isolates fell into clusters previously defined as dominant circulating clones (specifically *abscessus* DCC1 and *massiliense* DCC3) and are listed in Supplemental Table 1 (32).

### Analysis of *Mab* core and accessory genome content

**Fig 2:**
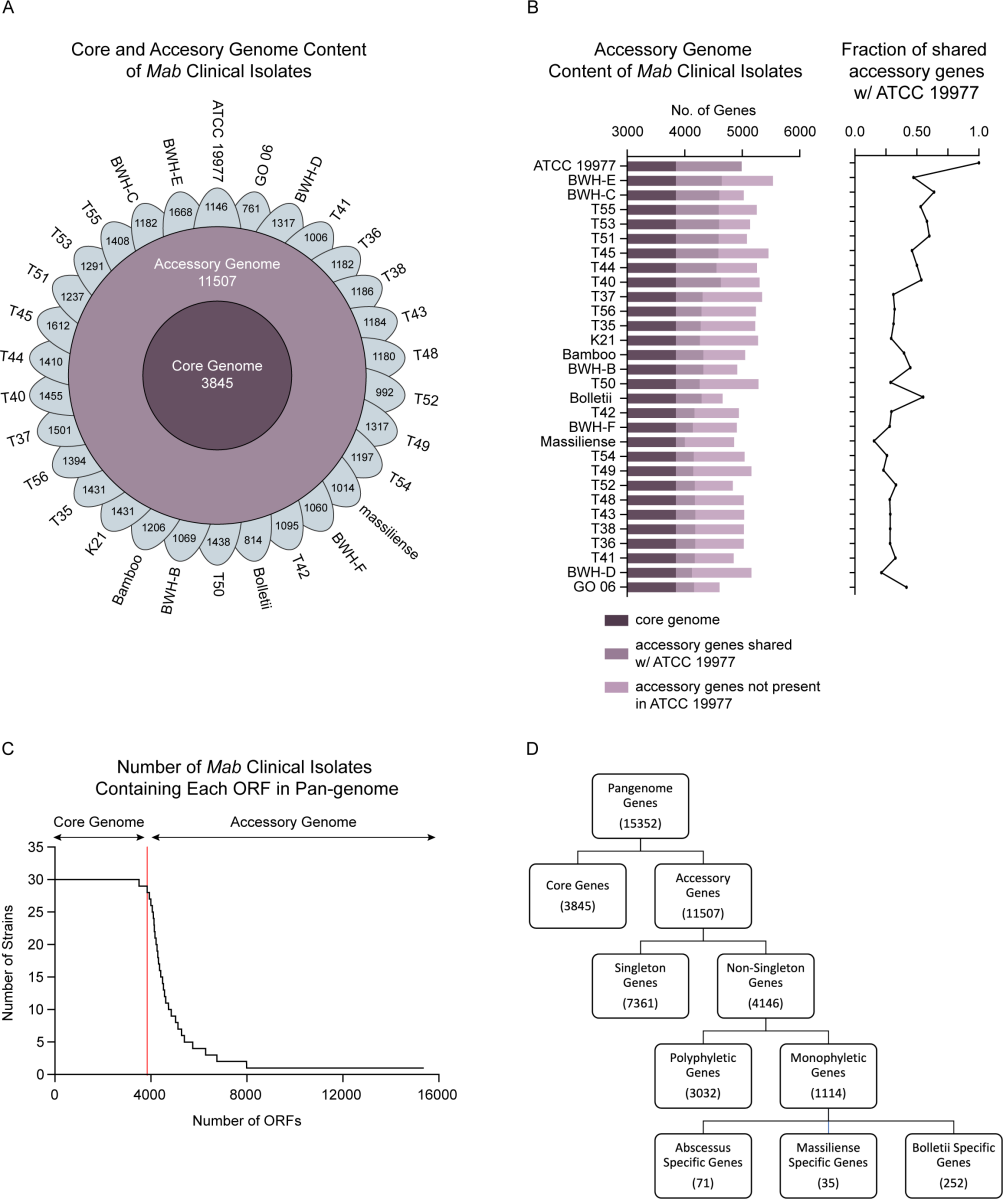
*Mab* has a large accessory genome. (**A**) Flower Pot diagram showing the breakdown of core and accessory genes in lab *Mab* clinical isolates. The outer leaflets represent the number of accessory genes in each clinical isolate. Strains are arranged counterclockwise based on the degree of similarity to the reference strain by the core genome. (**B**) (Left) Breakdown of accessory genome content in each clinical isolate. (Right) Percentage of accessory gene content shared by the reference strain. (**C**) Number of ORFs present in the clinical isolates. (**D**) Categorization of ORFs in the pan-genome.

Next, we identified the clinical isolates’ core and accessory genome content using the Ptolemy pan-genome analysis software program (33). From our panel of *Mab* clinical isolates, we identified 15,352 gene clusters representing distinct open reading frames (ORFs) (Table S3, https://orca2.tamu.edu/Mab_TnSeq/). Of these, ~25% of the gene clusters were in the core genome, defined as present in at least 95% of the 30 isolates. The remaining ~75% of gene clusters, or 11,507 ORFs, were in the accessory genome (Fig. 2A). The annotated genomes contained ~5,200 genes, with accessory genes ranging from 997 to 1,688. On average, the core genome comprised 75.6% of the total gene content in each strain, while the accessory genome accounted for 24.4%. These values are consistent with results published from previous studies (13, 34). As expected, isolates more distantly related to the reference ATCC 19977 strain generally shared less accessory genome content (Fig. 2B). The diversity of the accessory genome was remarkable, as evidenced by the number of isolates that contained distinct ORFs not present in any other isolates (Fig. 2C). Of the 11,507 accessory genes identified, nearly 64% (*n* = 7,361) were present in only one strain. The remaining 4,147 genes were present in multiple (2–28) isolates. Of these, 1,114 were monophyletic genes, meaning they were either inserted or deleted at one evolutionary time point and subsequently inherited by all the strains on that branch of the phylogenetic tree. The remaining 3,210 accessory genes were polyphyletic. A subset of the monophyletic genes was subspecies specific and present in only *abscessus, massiliense,* or *bolletii* isolates (Fig. 2D).

### Transposon-sequencing of multiple *Mab* clinical isolates

Given the large genomic diversity present in *Mab*, we performed TnSeq across multiple clinical isolates to characterize the species’ functional diversity. The goal was to identify genes that were differentially required among the isolates to better understand the functional diversity in *Mab*. Specifically, we sought to identify a core set of essential genes in *Mab* clinical isolates and genes whose essentiality was subspecies or lineage-specific. To do this, we selected 21 isolates for TnSeq, which spanned the phylogenetic tree of *Mab*, including 15 subsp. *abscessus* and six subsp. *massiliense*. We then generated libraries in triplicate for the *abscessus* subsp. isolates, and single replicate libraries for the *massiliense* subsp. isolates. A schematic of the TnSeq experiment is presented in Fig. S1A. The transduction efficiency of the clinical isolates varied greatly, with the highest efficiencies observed in the two reference strains: ATCC 19977 and *massiliense* CCUG 48898 (hereafter referred to as *massiliense*) (Fig. S1B). After transduction, >150,000 independent mutants were harvested from each replicate, and sequencing libraries were prepared for next-generation sequencing. Of note, subsp. *bolletii* libraries were not generated in this study due to the absence of clinical isolates in the lab.

Sequencing revealed that nearly all the transposon libraries were well-saturated, with a median saturation of 59.5% (“TA” dinucleotide sites containing a transposon insertion). This range of saturation spanned 20% to 70.3% (Fig. S1C). Libraries with less than 33% saturation were excluded from further analysis. Previous TnSeq studies demonstrated that downstream analyses of gene essentiality performed well on libraries with saturation between 30% and 80%. This analysis is also supported by the sensitivity analyses for the Hidden Markov Model (HMM), resampling, and analysis of variance (ANOVA) presented in the Supplemental Methods. The summary statistics for each library are presented in Table S4 (https://orca2.tamu.edu/Mab_TnSeq/). To assess the reproducibility of our replicates, we compared the mean number of insertions in each gene between pairs of replicate libraries for each strain. We found that most replicates were well correlated with a median Pearson r^2^ value of 0.88 (Table S5, https://orca2.tamu.edu/Mab_TnSeq/).

### HMM reveals essential genes in *Mab* clinical isolates

Using the HMM algorithm (35), we determined the essentiality of genes in each isolate. The genes were categorized as essential, non-essential, growth defect, or growth advantage. Essential genes have no insertions in all or most of their TA sites. Genes classified as “growth defect” are not essential, but their disruption causes growth impairment, resulting in suppressed transposon insertion counts. Conversely, when disrupted, genes classified as “growth advantage” cause a fitness benefit relative to wild type, resulting in increased insertion counts. The essential genes of the reference strain, ATCC 19977, have been previously reported (25, 27). We compared our predicted essential or growth-defect genes for ATCC 19977 to those in Rifat et al. (25) and found an overlap of 393 genes, showing high concordance between these two studies of *Mab* essentials (Fig. S2A). We also compared our predicted *Mab* essentials and growth-defect genes with those from the related mycobacterium *M. tuberculosis* (*Mtb* strain H37Rv) (36). There were 403 essential genes in common among 2,278 genes with mutual orthologs, demonstrating substantial conservation between the two mycobacterial species (Table S6, https://orca2.tamu.edu/Mab_TnSeq/). In total, there were 353 essential genes in the intersection of all three data sets (Fig. S2A). These genes were significantly enriched in key cellular pathways such as protein translation, amino acid metabolism, nucleotide metabolism, cell-cycle control, and cell wall synthesis (Fig. S2B). We then used the HMM to determine essential genes in all the clinical isolates. The essentiality call for each gene is listed in Table S7 (https://orca2.tamu.edu/Mab_TnSeq/). The average number of essential (ES) genes across the clinical isolates was 345, with a range of 281–391 (Fig. S3). The combined number of essential and growth-defect (ES+GD) genes (avg ~423) is consistent with essentiality analyses of other mycobacteria, where essential genes typically represent 10%–15% of the genome (27). There were 259 pan-essential genes that were ES or GD across all 21 Mab isolates. T These genes are listed in Table S7. Chi-squared analysis was used to identify 17 clade-specific essential genes (Fig. S4), such as *mqo* (MAB_3159c, malate:quinone oxidoreductase), which is only essential in subsp. *massiliense*.

### ANOVA identifies additional differentially required genes

Next, we identified differentially required genes, defined as genes whose disruption causes varying degrees of growth defect in isolates. Traditionally, the “resampling” method from TRANSIT is used to identify differentially required genes between two strains (35). We performed resampling between each clinical isolate and the reference ATCC 19977 strain, and the conditionally essential genes are listed in Table S8 (https://orca2.tamu.edu/Mab_TnSeq/). Resampling between pairs of isolates showed greater similarity (fewer conditionally essential genes) in comparisons within the same subspecies than those between subspecies (Fig. S5). These results gave us further confidence that our libraries were of sufficient quality for further analyses.

**Fig 3:**
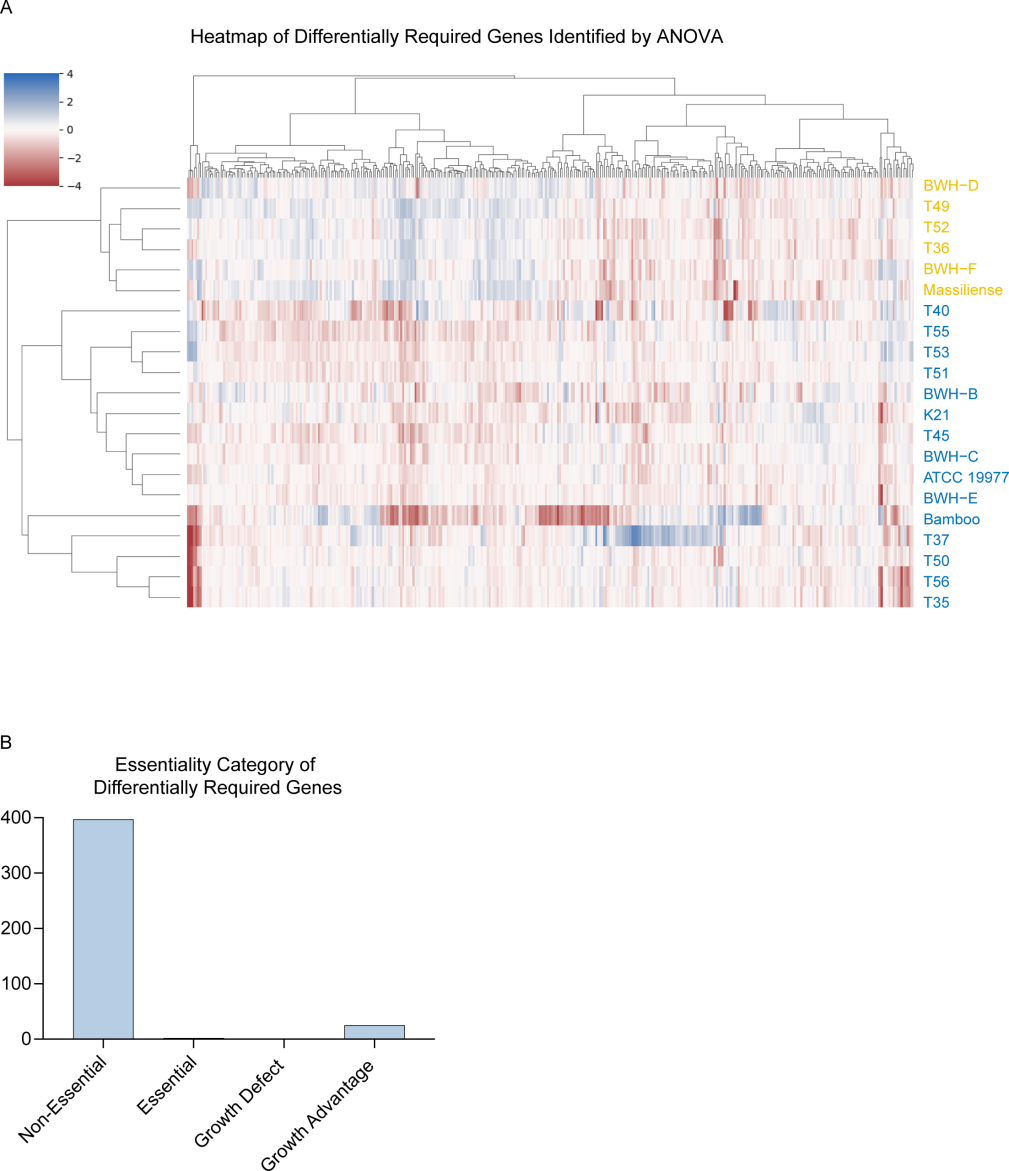
Summary of ANOVA results. (**A**) Heatmap depicting the LFC of insertion counts for the 425 differentially required genes in all isolates. (**B**) Essentiality category of the differentially essential genes in the ATCC 19977 reference strain.

To compare the transposon insertion counts across all strains simultaneously, we used ANOVA, which identified 425 genes in the pan-genome (with adjusted *P*-value < 0.05) that were differentially required across all 21 clinical isolates (Table S9, https://orca2.tamu.edu/Mab_TnSeq/). For each gene, the log_2_-fold-change (LFC) of the insertion counts in each isolate was calculated relative to the grand mean across all isolates. These LFCs are depicted in a heatmap (Fig. 3). Hierarchical clustering of the isolates revealed that the *massiliense* isolates (T36, T49, T52, BWH-D, BWH-F, massiliense) and the *abscessus* subspecies group together (Fig. 3). Thus, the phylogenetic tree generated from TnSeq data roughly recapitulates the phylogeny generated from SNP differences (Fig. 1A). The 425 differentially required genes represent 11% of the *Mab* core genome. Furthermore, 337 of these genes are non-essential in the reference ATCC 19977 genome (Fig. 3B). These data reinforce the concept that genes can be differentially required based on genetic background (24).

### Gene essentiality varies by lineage and strain

**Fig 4:**
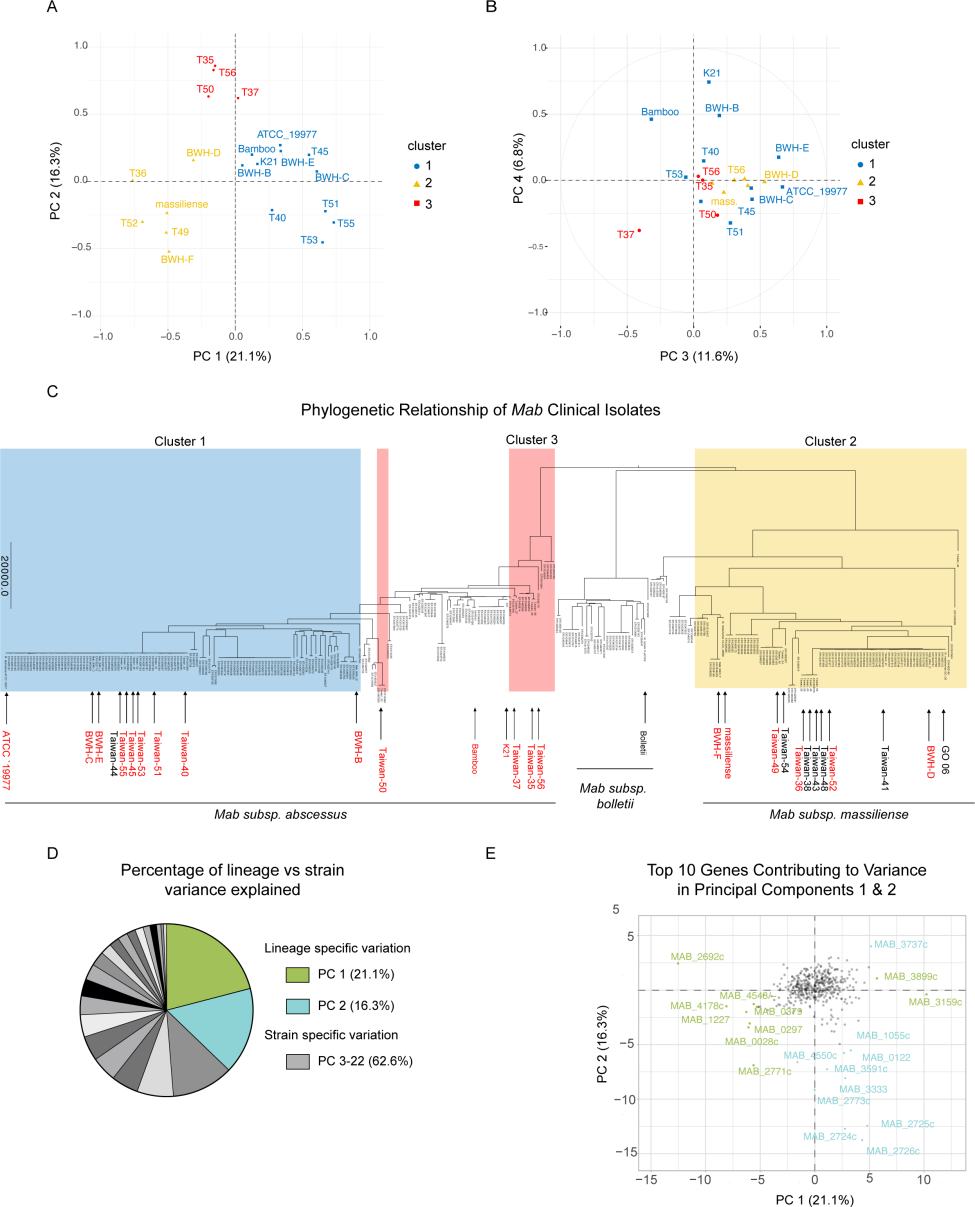
Principal component analysis of ANOVA hits. (**A**) PC 1 and 2 of differentially required genes by ANOVA. (**B**) PC 3 and 4. (**C**) Phylogenetic tree of *Mab* clinical isolates shaded with rectangles highlighting clusters. (**D**) Percentage of variance explained by each of the principal components. (**E**) Plot depicting the top 10 contributing genes to the variance of PC 1 and 2.

To better understand the variance in the ANOVA data set, we used principal component analysis (PCA) to group the significant genes. The analysis showed principal components (PC) 1 and 2 explain ~40% of the variation in the data (Fig. 4A). When the isolates are clustered into three groups, they do so primarily by phylogenetic relationship, where cluster 1 is a sub-lineage of the *abscessus* subspecies, cluster 2 has the *abscessus* subspecies that are more closely related to the reference ATCC 19977 strain, and cluster 3 contains the *massiliense* subspecies. PCs 1 and 2 represent lineage-specific differences in gene requirement, while when PCs 3 and 4 are plotted, the phylogenetic clustering collapses (Fig. 4B). The *Mab* phylogenetic tree is color-coded with the three corresponding clusters (Fig. 4C). This analysis demonstrates that closely related isolates share genetic requirements and suggests a shared evolutionary origin of how these requirements arose.

The variation in PCs 1 and 2 only explains ~40% of the variation in the data set, corresponding to lineage-specific variation. In contrast, PCs 3–19 explain the remaining ~60% of the variation and likely represent strain-specific variation or noise (Fig. 4D). The top 10 genes contributing to the variance in PCs 1 and 2 are plotted in Fig. 4E. The genes in PC 1 separate the two subspecies, while those in PC 2 separate Cluster 1 strains (T35, T37, T50, and T56) from the rest of the clinical isolates (Fig. 4E).

### Identification of genes with lineage-specific essentiality

**Fig 5:**
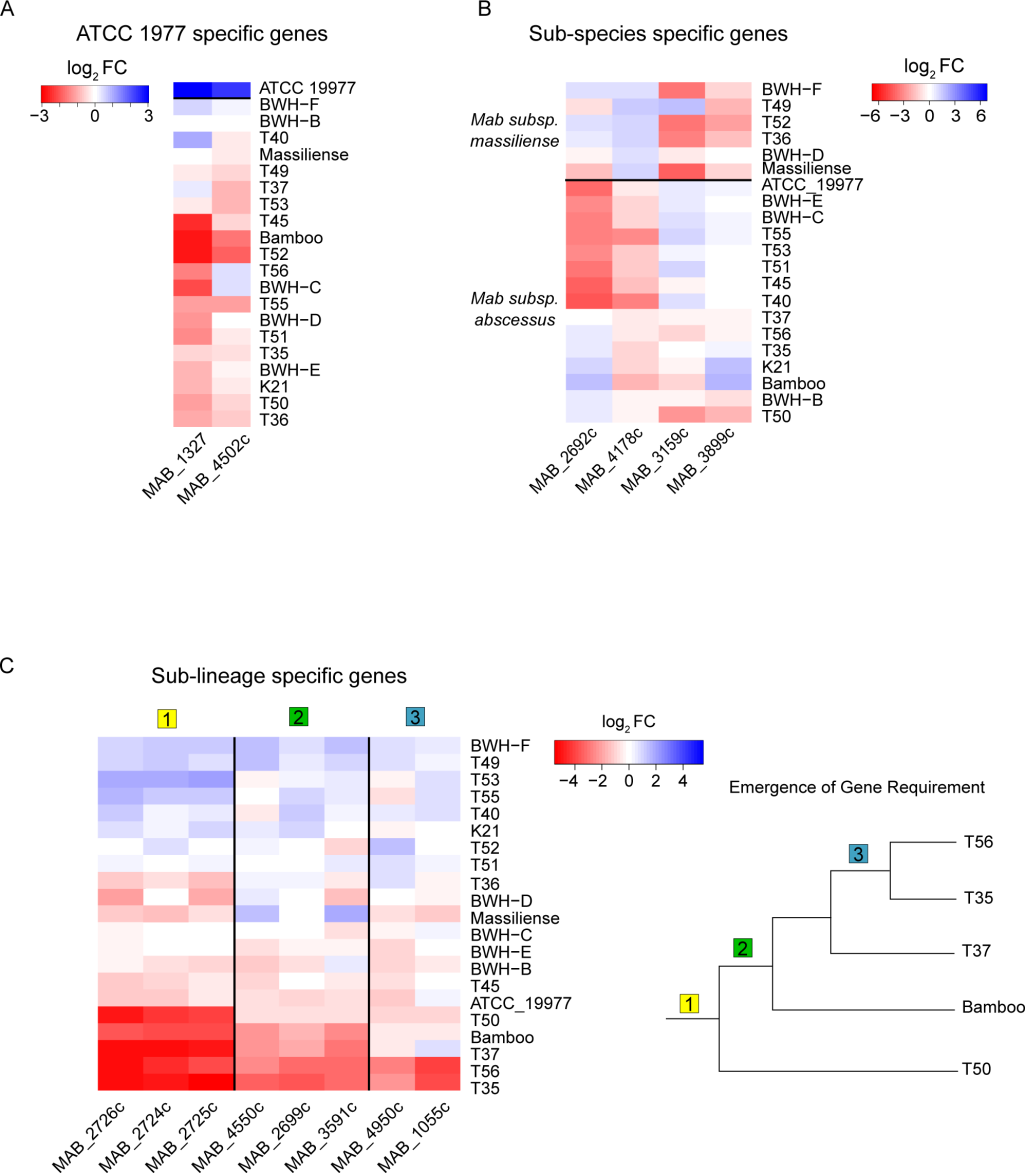
Subspecies and lineage-specific gene requirement. (**A**) Genes specific to the reference ATCC 19977 strain. (**B**) Subspecies-specific genes that separate *abscessus* and *massiliense* subspecies. (**C**) (Left) Genes that are differentially required in cluster 1. (Right) Schematized phylogenetic relationship of strains in cluster 1.

Next, we examined genes that exhibited lineage-specific differences in genetic requirement. We first identified genes that separated the reference strain from the clinical isolates. Two genes were the strongest differentiators: *MAB_4502*c, which encodes phosphoenolpyruvate carboxykinase (*pckA*), and *MAB_1327,* which encodes ferredoxin (*fdxC*) (Fig. 5A). The reference strain requires *pckA* less than the clinical isolates, a difference that could be explained by laboratory adaptation of the reference strain.

Next, we analyzed the contributing genes in PC 1 that separate the two subspecies of *Mab abscessus* vs *massiliense*. The top four genes are depicted in Fig. 5B, while the top 20 genes that separate the subspecies are listed in Table S10 (https://orca2.tamu.edu/Mab_TnSeq/). We next inspected genes that separated strains in Cluster 1 (T35, T37, T50, and T56) from the rest of the clinical isolates (Fig. 5C). The top 20 genes contributing to the variation in this cluster are listed in Table S11 (https://orca2.tamu.edu/Mab_TnSeq/).

The genes uniquely required in Cluster 1 can be split into three groups depending on how many strains share the level of gene requirement. For group 1, all four strains in the cluster share the same requirement for three genes in an operon, *MAB_2724c-MAB_2726*c (hypothetical genes of unknown function). Group 2 genes are shared only by T35, T37, and T56, which are more related to each other than T50. Finally, group 3 comprises genes shared by the two most closely related strains, T35 and T56. These data reveal genetic requirements emerging within a lineage. As strains diverge, their genomes change in genetic sequence and function, and genes that are otherwise dispensable in one strain can become more required in another. This is best illustrated by genes *MAB_4950*c and *MAB_1055*c, which are most strongly required in the two phylogenetically related isolates, T35 and T56 (Fig. 5C).

### Correlated transposon insertion counts reveal functional genetic networks

**Fig 6:**
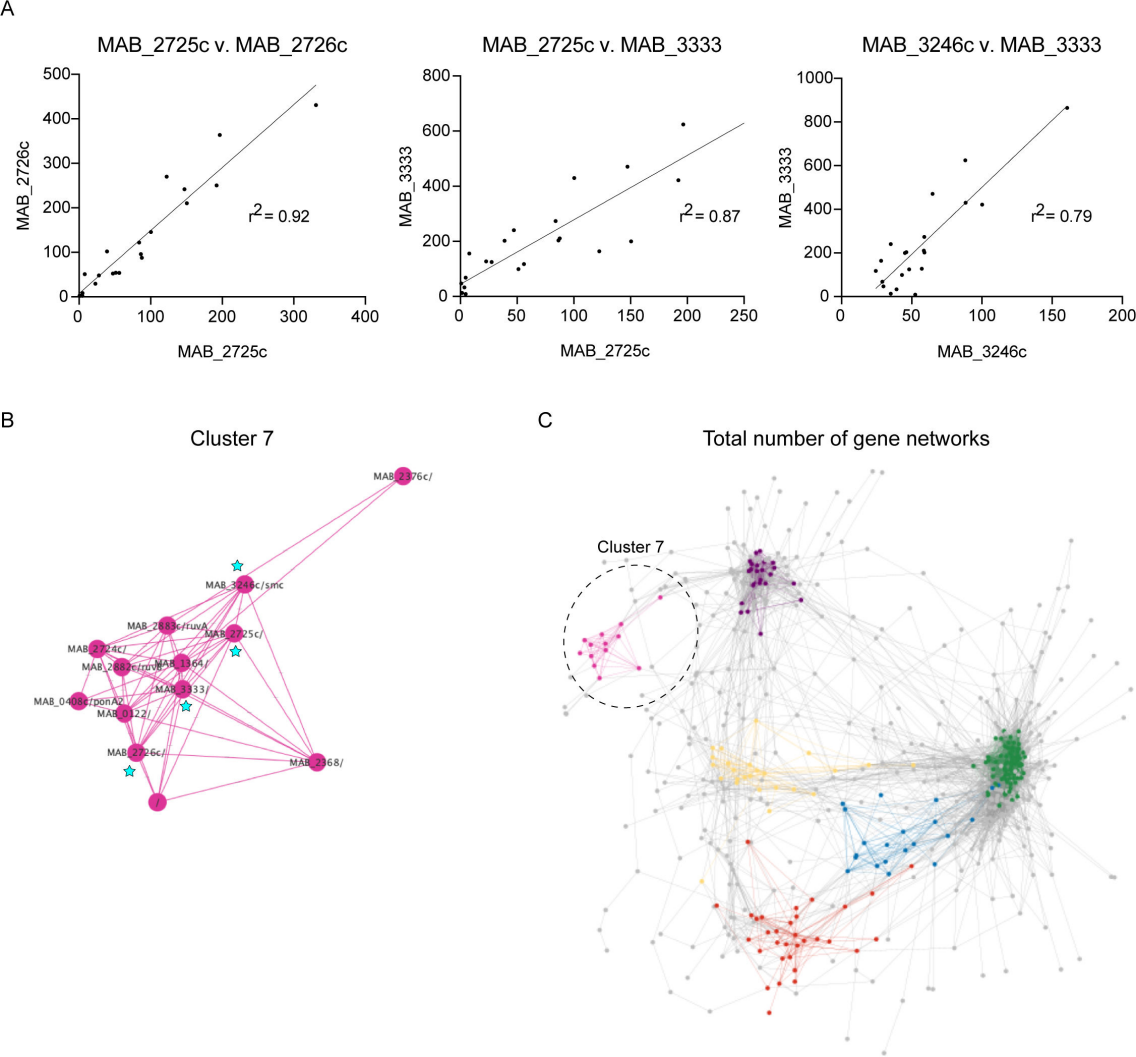
Network analysis of differentially required genes. (**A**) Correlation of transposon insertions between three gene pairs. (**B**) Cluster 7 depicts 13 genes that are correlated with each other. Genes marked with blue stars are shown in panel A. (**C**) Network analysis depicting five additional clusters of 19 identified. Cluster 7 is highlighted by a dashed circle.

To extract functional inferences from our TnSeq data set, we looked for pairs of genes with correlated transposon insertion counts, which could reveal hidden functional genetic networks. To conduct this analysis, we selected the 751 unique ORFs with the most variability based on ANOVA (*P*-adj < 0.05) and other filters (see Materials and Methods). Of the 282,000 potential gene pairings, only 10,198 were significantly correlated, representing 3.6% of total potential pairs (Table S12, https://orca2.tamu.edu/Mab_TnSeq/). In Fig. 6A, we show three examples of gene pairs that are positively correlated. Pair 1 is genes *MAB_2725c x MAB_2726*c, whose insertion counts were correlated with an r^2^ of 0.92. The following two pairs, *MAB_2725c x MAB_3333* and *MAB_3246c x MAB_3333,* also have statistically significant correlated insertion counts. All these genes are correlated with each other and ostensibly form a network.

Based on these pairwise gene correlations, we performed network analysis with Cytoscape to turn these pairwise correlations into a network of interconnected genes. The network was organized into 19 clusters based on a greedy community identification algorithm (see Materials and Methods), so related genes with similar transposon insertion profiles were clustered. This analysis identified 19 clusters containing at least five genes (Table S13, https://orca2.tamu.edu/Mab_TnSeq/). The genes profiled in Fig. 6A are members of cluster 7 and are starred in the network (Fig. 6B). Cluster 7 is comprised of 13 genes and includes the previously highlighted operon *MAB_2724c–MAB_2726* as well as 10 other genes, including *ruvA* and *ruvB*, which both encode Holliday junction ATP-dependent DNA helicases, and *MAB_3246*c, which encodes *smc*, segregation and condensation protein (Fig. 6B). The complete network analysis with a few of the clusters highlighted is depicted in Fig. 6C. Importantly, only comparative analysis of TnSeq data could reveal these genetic networks across multiple genetically diverse strains.

### Genes involved in central carbon metabolism are differentially required among clinical isolates

**Fig 7:**
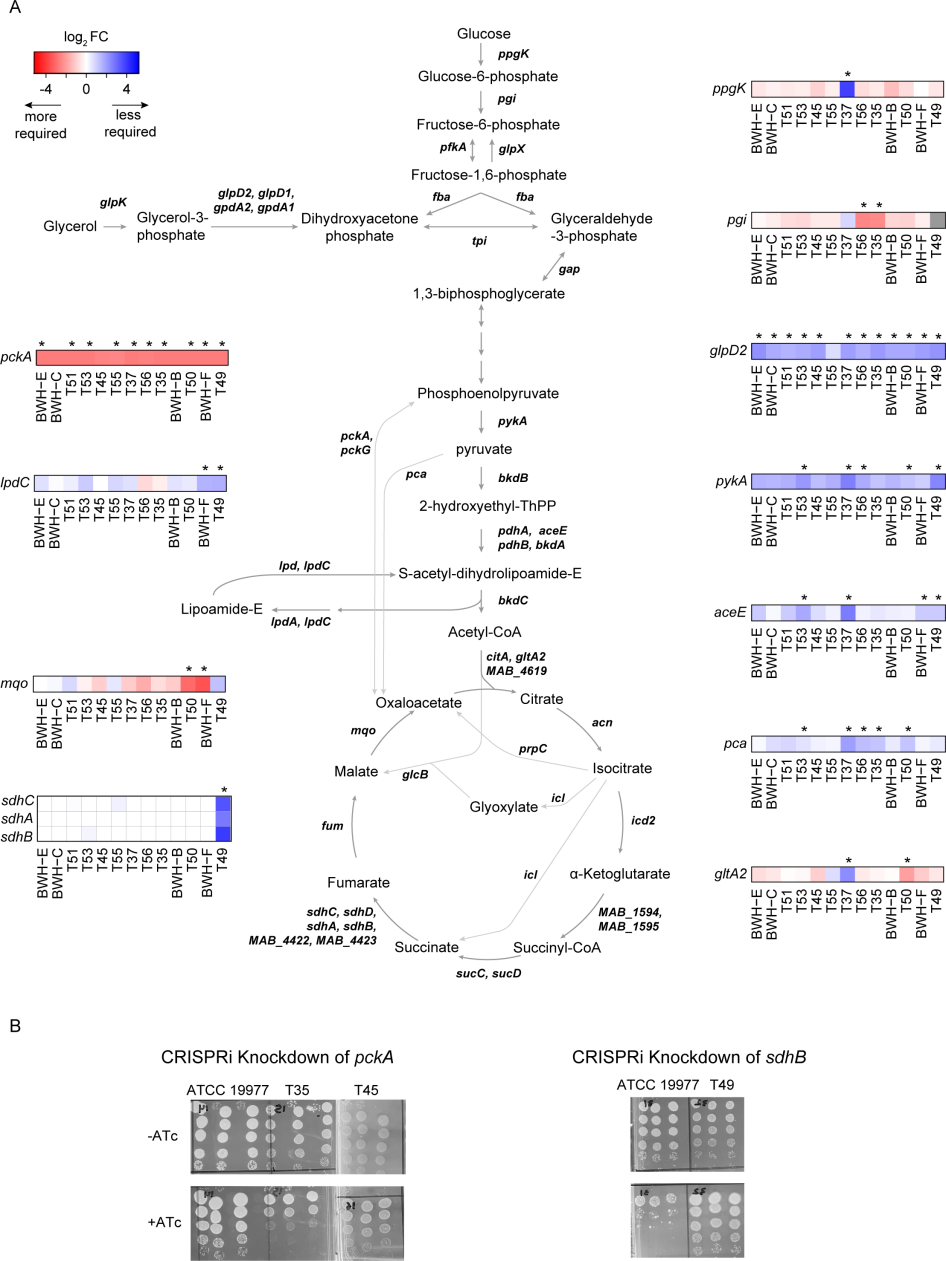
Genetic requirement differences in carbon metabolism genes among clinical isolates. (**A**) Schematic of genes in central carbon metabolism in *Mab*. All heatmaps showcase LFC relative to the reference strain. Genes with significant differences in LFC are highlighted with an “*” indicating a *P*-adj < 0.05. (**B**) CFU assay of *pckA* or *sdhB* clinical isolate knockdowns.

Resampling analysis unveiled several differentially required genes involved in carbon metabolism among the clinical isolates compared to the reference strain (Fig. 7A). As noted above, *pckA* is more required in clinical isolates than ATCC 19977. Other examples include *mqo* (malate:quinone oxidoreductase), which is most required in T50 and BWH-F, and *pgi* (glucose-6-phosphate isomerase), which is most required in T35 and T56. To validate these differences, we employed CRISPRi/Cas9 to repress *pckA*, which is more required in nearly all the clinical isolates and is dispensable in ATCC 19977. We found that when *pckA* is repressed in ATCC 19977, there was no difference in CFUs between induction and non-induction plates as predicted by the TnSeq data (Fig. 7B). However, when we repressed the enzyme in T35 and T45, we observed a 10× to 100× decrease in CFUs, indicating that *pckA* is more required in these clinical isolates. Notably, *pckA* was significantly more repressed in the reference strain than in the clinical isolates (Fig. S6A). The lack of reduction in CFUs in the reference strain despite stronger knockdown further supports *pckA’s* dispensability in ATCC 19977 relative to the tested clinical isolates.

We also repressed *sdhB*, which was predicted to be required in ATCC 19977 and less required in the T49. Repressing *sdhB* in ATCC 19977 led to a 100× fold decrease in CFUs. However, when *sdhB* was repressed in T49, there was no difference in CFUs, confirming that *sdhB* is less required in T49 (Fig. 7B). These differences in CFUs were observed with similar levels of *sdhB* knockdown (Fig. S6B). Altogether, these knockdown experiments validate our genetic requirement data and corroborate the functional differences in carbon metabolism between the clinical isolates.

## DISCUSSION

While several groups have described the genomic diversity of *Mab* clinical isolates (13, 16, 17), to date, no studies have interrogated the functional consequence of this diversity. To investigate the functional diversity and genetic requirements of *Mab*, we performed TnSeq on 21 *Mab* clinical isolates spanning both the *abscessus* and *massiliense* subspecies. Among the 21 isolates, we identified 425 genes, or ~11% of the *Mab* core genome as being differentially required among the isolates. This includes non-essential genes in the reference strain that became either fully essential or more required in some clinical isolates.

Applying TnSeq to a genetically diverse group of isolates enabled us to uncover functional differences among core and accessory genes. Using PCA and network analysis, we identified significant clusters of genes whose insertion patterns were correlated and whose functional requirements varied with phylogenetic position, either subspecies or lineage-specific. This data implied that *Mab* isolates have evolved to require a distinct subset of genes. Given that our genetic screen was conducted under *in vitro* conditions, passaging our transposon-mutant libraries through more environmental or disease-relevant conditions would reveal additional variability in genetic requirement. Recently, Sullivan et al. passaged a transposon-mutant library of clinical isolate T35 on an *in vitro* lung model and identified key genes responsible for establishing lung infection (37).

We also identified differences in gene requirements that were lineage-specific. Strains T35, T37, T50, and T56 represent one distinct lineage of the *abscessus* subspecies with genetic requirements unique to these strains. Notably, the operon *MAB_2724c–MAB_2726*c was uniquely required in this lineage and contributed 20% of the variance separating this sub-lineage from all the other clinical isolates. All three genes in the operon are annotated as hypothetical proteins (orthologous to Rv1479-81 in *Mtb*), and future studies are needed to understand why this operon is uniquely required in this subset of strains.

Interestingly, our study identified functional differences between the reference strain and the clinical isolates in genes related to carbon metabolism. Specifically, the gene *MAB_4502*c encodes the enzyme phosphoenolpyruvate carboxykinase, *pckA*, and is more required in nearly clinical isolates than the reference strain. *pckA* catalyzes the conversion of oxaloacetate to phosphoenolpyruvate, the first committed step of gluconeogenesis. The reduced requirement for *pckA* in ATCC 19977 is likely a consequence of the reference strain passaged on rich media and adapting to ample carbon sources for 70+ years (38).

This study raises an open question: what role does the accessory genome play in driving variability in genetic requirements among *Mab* strains? *Mab* is well-known to have a large accessory genome, which we confirmed using pan-genome analysis. Gene acquisition and gene loss through plasmid transfer, phage integration, duplication, or large-scale deletion likely influences genetic requirements and functional differences between strains. More work is needed to untangle the effects of these accessory genes’ presence and absence on the core genome’s essentiality. Nevertheless, this TnSeq data set offers valuable insight into variable functionality among *Mab* strains and provides a foundation for future study of the biological and clinical consequences of genetic diversity of *Mab* clinical isolates.

## MATERIALS AND METHODS

### Bacterial strains and culture conditions

*Mab* clinical strains were isolated from patients in Taiwan (T) and Brigham and Women’s Hospital (BWH), Boston, MA. Clinical isolates were streaked from patient stabs; single colonies were picked, cultured, and frozen for future use. Strains with transformed CRISPRi/Cas9 vectors to knockdown central carbon metabolism genes were built as described in reference (27). All strains were grown shaking at 37°C in liquid 7H9 media consisting of Middlebrook 7H9 salts with 0.2% glycerol, 0.85 g/L NaCl, and OADC (oleic acid, 5 g/L albumin, 2 g/L dextrose, 0.003 g/L catalase), and 0.05% Tween80. All *Mab* strains were plated on Middlebrook 7H10 agar supplemented with 10% OADC and 0.05% Tween80. Kanamycin 100 µg/mL was used for selection. All CRISPRi/Cas9 plasmids were induced with 500 ng/µL ATc.

### Whole-genome sequencing of *Mab* clinical isolates

Genomic DNA from late-log phase cultures were extracted via a bead-beating and phenol-chloroform protocol described in Akusobi et al. (27), using an Illumina Rapid HiSeq 2500 instrument with 125+125 bp paired-end reads.

### Genome assembly

Genome sequences for each isolate were assembled *de novo* from .fastq files using ABySS (v2.0) with default parameters (29). The assemblies had a mean total length of 5.14 Mbp (range: 4.86–5.54 Mbp) (Table S2). To assemble core genomes, the contigs were mapped onto the *M. abscessus* ATCC 19977 (NC_010397.1) reference genome using Blastn and concatenated in syntenic order. The final genome length and depth of coverage are shown in Table S2. To distinguish among *Mab* subspecies, the pattern of nucleotide substitutions was examined in *hsp65/groEL2* and rRNA (internal-spacer) loci (39).

### Genome alignment and annotation

Genome alignments were made using MUMMER v3.23 (40). To identify SNPs and indels, regions with genetic variation were aligned using the Needleman-Wunsch algorithm (41). For core genomes, the coordinates of ORFs were adjusted for each isolate using a whole-genome alignment to the reference genome. Genes whose start or end coordinates were deleted in a clinical isolate were removed from the annotation. To produce unbiased annotations of each clinical isolate, we ran RAST (PATRIC CLI v1.039) on the contigs generated by ABySS (42). These annotations contain all predicted ORFs on all contigs.

### Phylogenetic analysis

An alignment of all the core genomes was first generated to construct a phylogenetic tree incorporating all the *Mab* clinical isolates and reference strains. The included reference strains are listed in Table S2, with sequencing data for *M. abscessus* str. K21 kindly provided by M. Gengenbacher. For all isolates, regions with insertions or deletions were filtered out, along with sites exhibiting low coverage (<10×), heterogeneity (<70% base-call purity), or repetition (where an overlapping window of 35 bp matches with at least 33/35 bp elsewhere in the genome). Finally, the remaining list of SNPs was extracted and used as input to RAxML (43) to generate the maximum likelihood phylogenetic tree, rooted using *M. bolletii* str. FLAC003 as an outgroup. Gubbins (30) was used to identify and filter out genomic regions (at least 500 SNPs) potentially affected by recombination, totaling <15% of the genome.

### Pan-genome analysis

The pan-genome analysis was performed on 30 Mab strains (25 clinical isolates and five reference strains) using Ptolemy v1.0 (33) to determine mappings of orthologs among ORFs based on homology and synteny. ABySS assemblies were input for each isolate and the ORFs from the RAST annotation. The output was used to generate a spreadsheet with all “pan-ORFs” (15,352 ortholog clusters) as rows and individual ORF IDs in columns. This data is provided in Table S3, which also contains a count of how many of the 30 strains had a copy of each pan-ORF, which was used to differentiate core genes (genes with orthologs in >95% of isolates) from accessory genes (present in 1–28 isolates).

### Generation of transposon libraries

High-density transposon libraries of selected *Mab* clinical isolates were generated using the ϕMycoMarT7 phage described in Akusobi et al. (25–27). Libraries were generated in biological triplicate for 15 clinical isolates, while single-replicate libraries were generated for six isolates (see Table S4). K21 was a generous gift from Thomas Dick, Meridian Health, Hackensack, NJ.

Libraries were selected for 4 days on 7H10 agar supplemented with 10% OADC, 0.5% glycerol, 0.05% Tween80, and 100 µg/mL kanamycin. Libraries of >150,000 mutants were harvested and stored in 7H9 + 10% glycerol at −80°C for future use. Transposon-sequencing libraries were prepared following the protocol described in (44). The TnSeq samples were sequenced on an Illumina Rapid HiSeq 2500 instrument with 125 + 125 bp paired-end reads.

### TnSeq analysis

The .fastq files obtained from sequencing were pre-processed using TPP in Transit (35). The reads were then mapped to the syntenic versions of each genome (aligned to ATCC 19977) and the raw ABySS assemblies (all contigs) of each isolate. The median library saturation was 58.4% (19.0%–72.6%) (Table S4). BWH-B-2, BWH-C-1, and all T44 strains were excluded from further analyses due to low saturation or total insertion count.

The HMM in Transit was run to identify essential and non-essential genes in each isolate (Table S7). Furthermore, the genes were evaluated by a confidence score based on the consistency of the insertion statistics with the posterior distribution of the called essentiality state (see Supplemental Methods). Low-confidence genes (<0.20) were filtered out of the analyses. The “resampling” method in Transit was used to identify conditionally essential genes in each isolate relative to ATCC 19977. The *P*-values were adjusted for multiple tests using the Benjamini-Hochberg procedure (45) for an FDR of 5%. Significant genes are those with *P*-adj < 0.05 (Table S8). ANOVA analysis was then applied to the pan-ORFs to identify genes with significant variability of insertion counts across strains. The normalized counts at each TA site for each gene were tabulated and averaged at the strain level, excluding genes with mean <20. Then one-way ANOVA was used to calculate the F-statistic and *P*-value, adjusted for multiple testing as above.

### Genetic interactions and network analysis

A correlation analysis of mean transposon insertion counts was performed for gene pairs across all *Mab* strains. Genes with significant variability by ANOVA (*P*-adj < 0.05) and insertion count <20 or Z-max > 8 (used to filter out outliers) were selected, resulting in 751 varying genes. A Pearson correlation coefficient was calculated for gene pairs that were jointly present in at least 10 strains. This analysis identified 10,198 pairs of genes with a correlation of >0.7 and adjusted *P*-value < 0.05. A network diagram based on these correlations was generated in Cytoscape v3.9.1 using the Edge-weighted Spring-embedded Layout.

To extract clusters of genes with similar TnSeq profiles, a network community analysis was performed based on a greedy algorithm. The degree of each gene was calculated, reflecting the number of genes correlated to it with cc > 0.7. The gene with the highest degree represented the most “central” gene in the network and was required to be present in at least 15 strains. We extracted the cluster of genes connected to the most central gene as the first cluster. To extract subsequent clusters, we identified the next most central gene whose neighbors did not overlap more than 20% with genes in previously selected clusters. The process was iterated until clusters contained fewer than five genes.

### RNA extraction and RT-PCR

For each strain, cultures were grown in biological triplicate to mid-log phase, diluted back in ±500 ng/mL ATc, and grown for 18 h to achieve target knockdown. Afterward, 2 OD_600_ equivalents of cells from each culture were harvested by centrifugation, resuspended in TRIzol (Thermo Fisher), and lysed by bead beating (Lysing Matrix B, MP Biomedicals). RNA extraction and subsequent reverse transcription PCR (RT-PCR) experiments were performed as described by Akusobi et al. (27).

## SUPPLEMENTAL MATERIAL

Figure S1 mbio.03376-24-s0001.tif Summary of TnSeq data.

Figure S2 mbio.03376-24-s0002.tif Comparison of *M. tuberculosis* and *M. abscessus* essential genes.

Figure S3 mbio.03376-24-s0003.tif Summary of gene essentiality category across *Mab* clinical isolates.

Figure S4 mbio.03376-24-s0004.tif Analysis of shared essential genes in *Mab* clinical isolates.

Figure S5 mbio.03376-24-s0005.tif Pairwise resampling heatmap matrix.

Figure S6 mbio.03376-24-s0006.tif Fold repression of *pckA* and *sdhB*.

Supplemental Methods mbio.03376-24-s0007.docx Functional analysis of conserved essential genes, confidence assessment of essentiality calls by HMM, sensitivity analysis, and plasmids.

Supplemental Legends mbio.03376-24-s0008.docx Legends for Fig. S1 to S6.

ASM does not own the copyrights to Supplemental Material that may be linked to, or accessed through, an article. The authors have granted ASM a non-exclusive, world-wide license to publish the Supplemental Material files. Please contact the corresponding author directly for reuse.
