# Supplementary figures and images for: Transposon-sequencing across multiple Mycobacterium abscessus isolates reveals significant functional genomic diversity among strains
Source: mBio. 2024 Dec 31;16(2):e03376-24. doi: 10.1128/mbio.03376-24 (PMC11796383; doi:10.1128/mbio.03376-24)

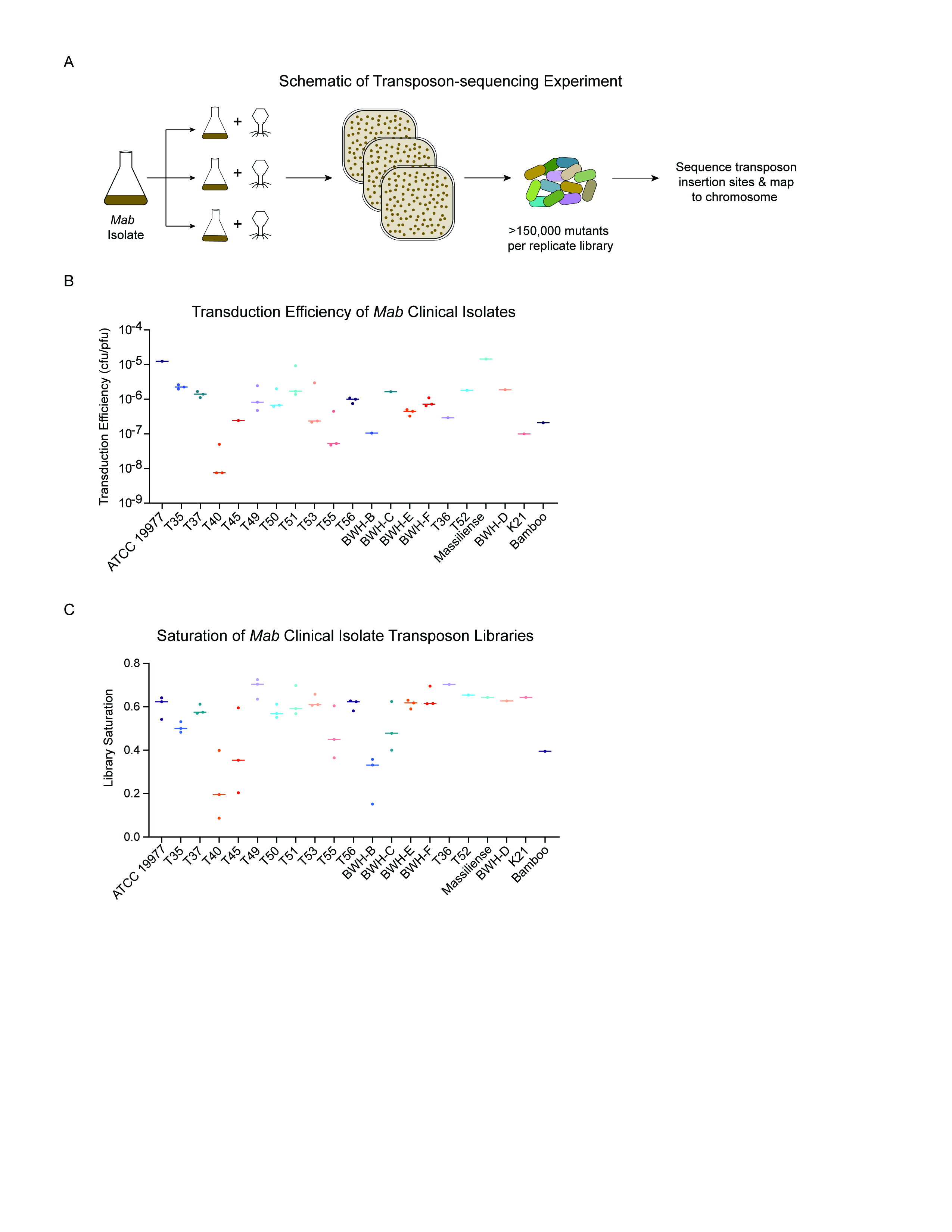

Supplement: Figure S1 — Summary of TnSeq data. [file mbio.03376-24-s0001.tif]

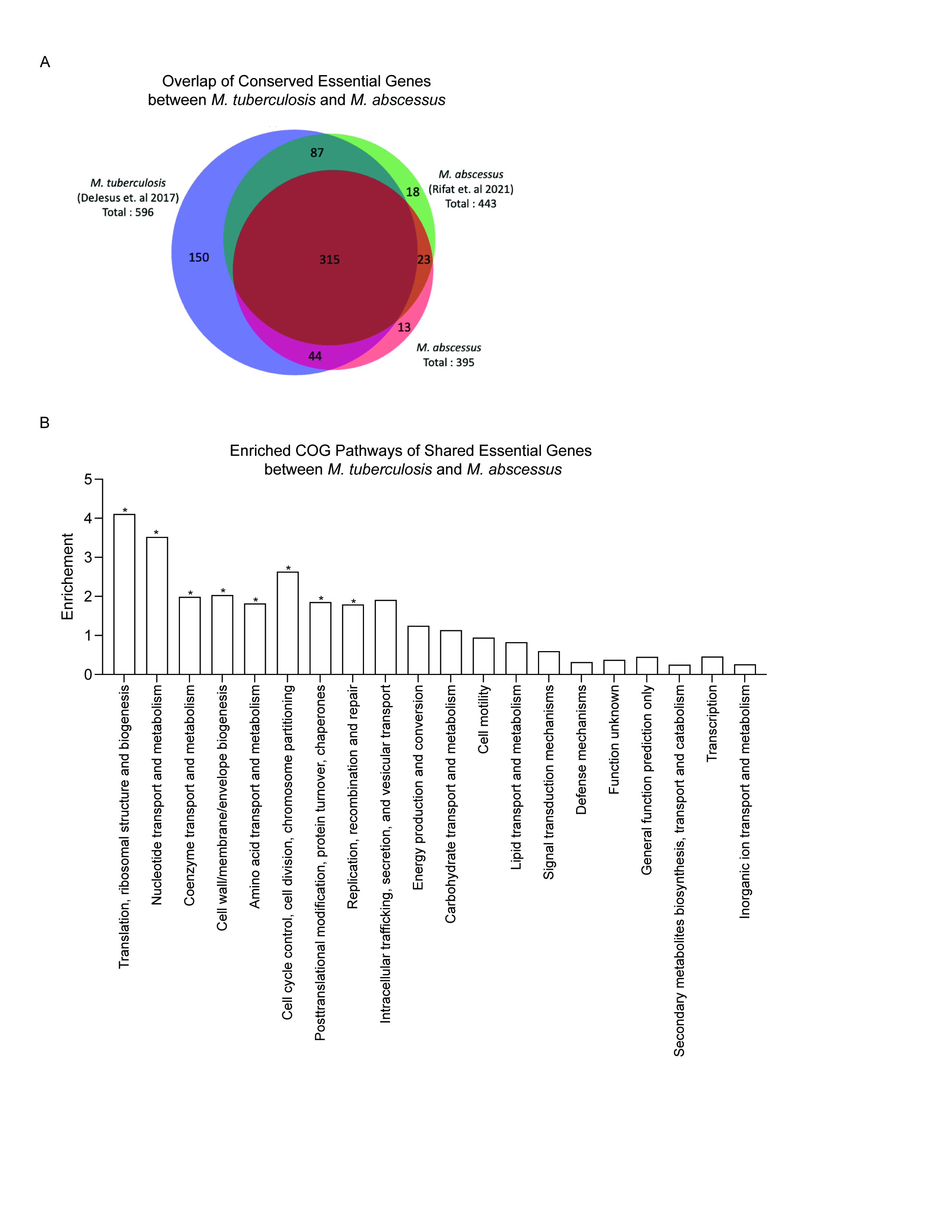

Supplement: Figure S2 — Comparison of M. tuberculosis and M. abscessus essential genes. [file mbio.03376-24-s0002.tif]

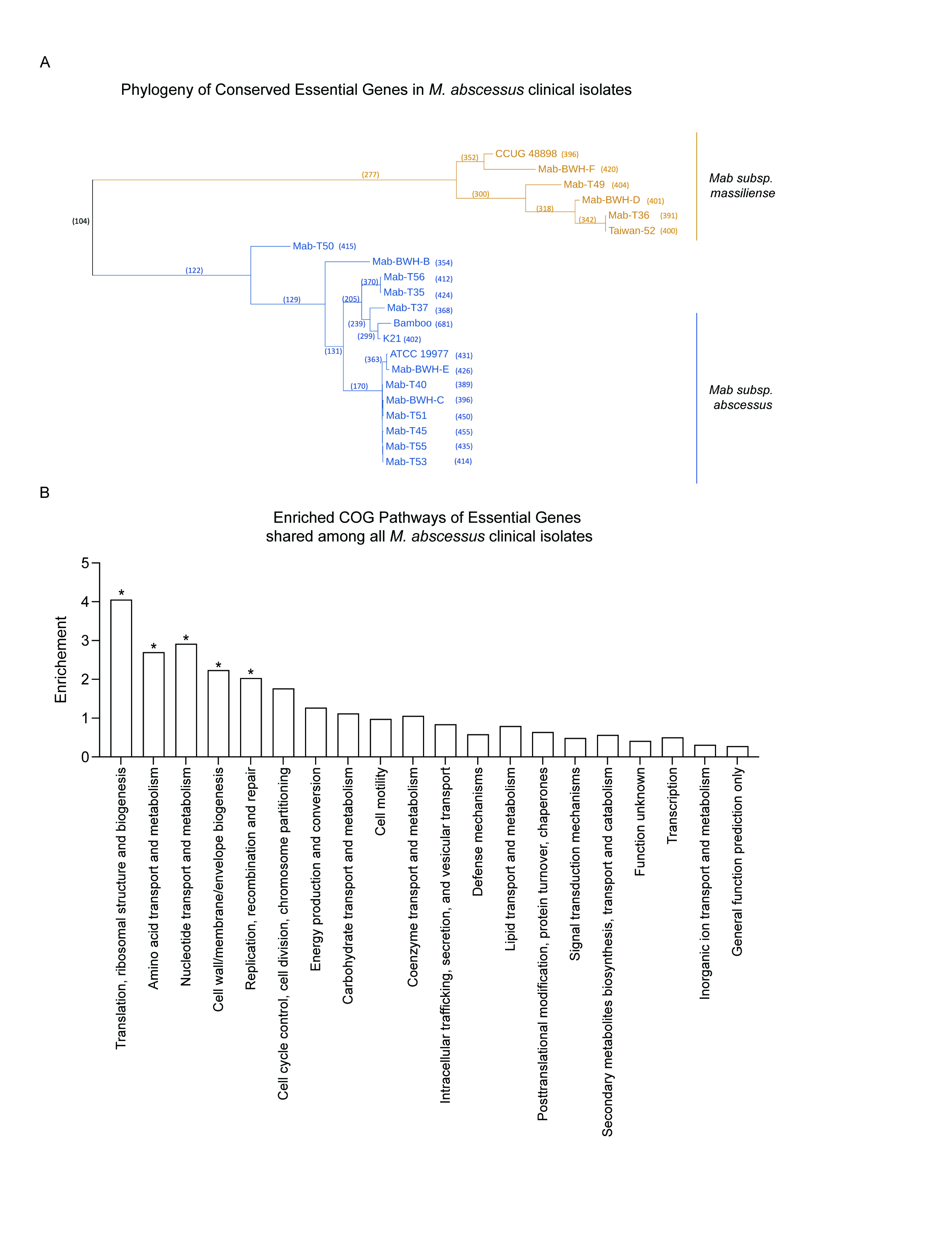

Supplement: Figure S3 — Summary of gene essentiality category across Mab clinical isolates. [file mbio.03376-24-s0003.tif]

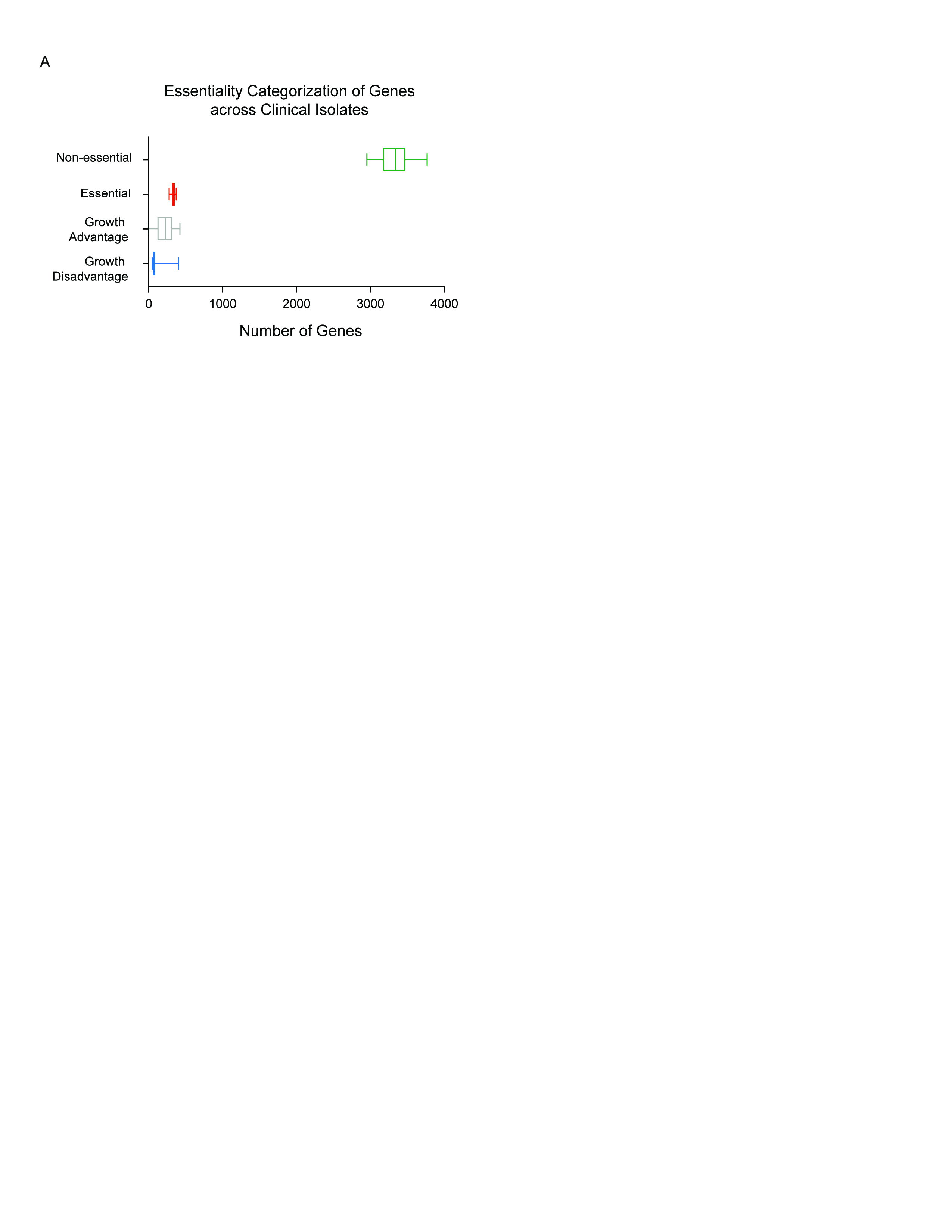

Supplement: Figure S4 — Analysis of shared essential genes in Mab clinical isolates. [file mbio.03376-24-s0004.tif]

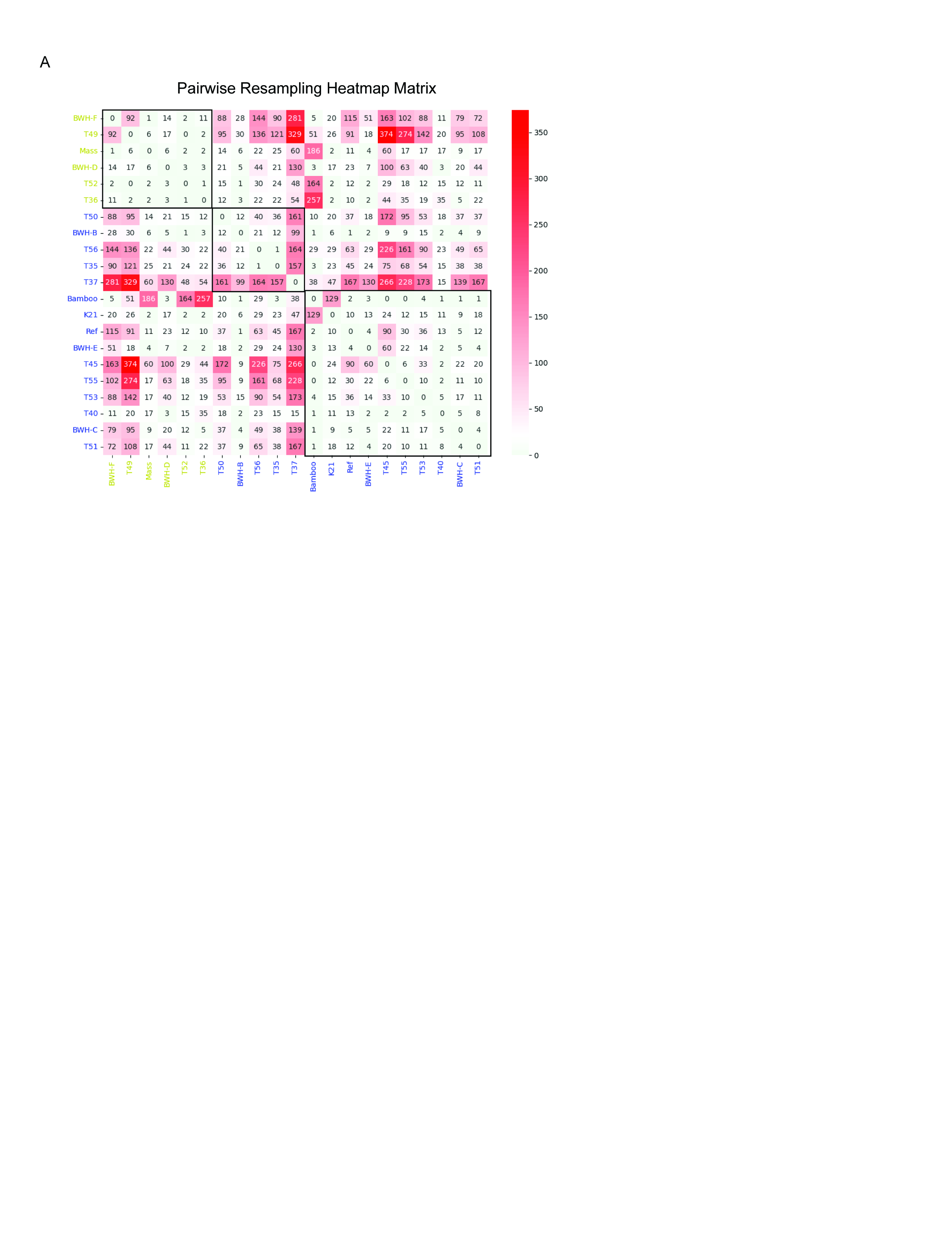

Supplement: Figure S5 — Pairwise resampling heatmap matrix. [file mbio.03376-24-s0005.tif]

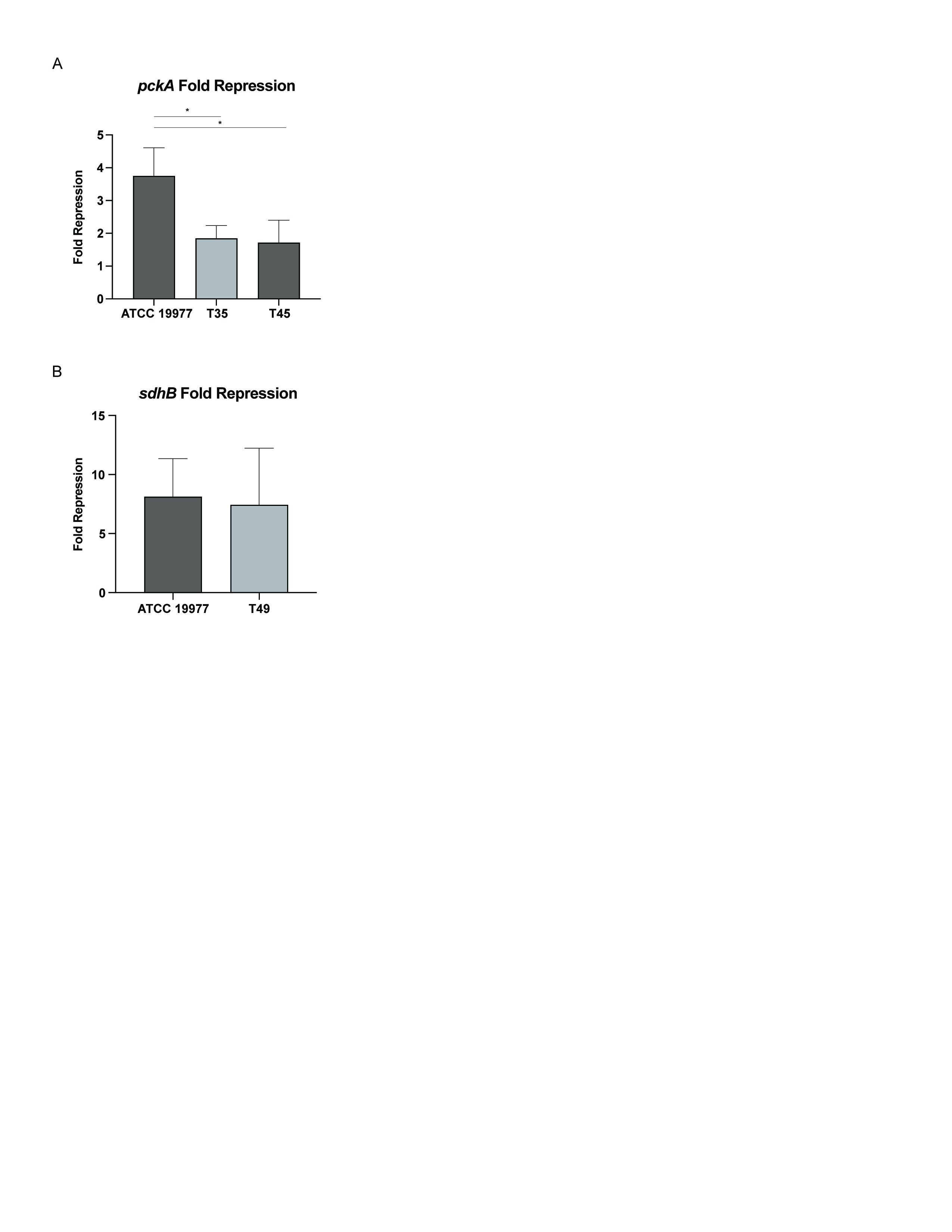

Supplement: Figure S6 — Fold repression of pckA and sdhB. [file mbio.03376-24-s0006.tif]
