# Supplementary material for: Transposon-sequencing across multiple Mycobacterium abscessus isolates reveals significant functional genomic diversity among strains
Source: mBio. 2024 Dec 31;16(2):e03376-24. doi: 10.1128/mbio.03376-24 (PMC11796383; doi:10.1128/mbio.03376-24)
Supplement: Supplemental Legends — Legends for Fig. S1 to S6. [file mbio.03376-24-s0008.docx]

**Supplemental Legends**

**Supplemental Figure 1: Summary of TnSeq data. (A)** Schematic of TnSeq experiment. **(B)** Transduction efficiency (CFU/PFU) of *Mab* clinical isolates. (**C)** Percent saturation of TnSeq libraries. The dotted line demarcates 30% library saturation used as a threshold for study inclusion.

**Supplemental Figure 2: Comparison of *M. tuberculosis* and *M. abscessus* essential genes. (A)** Overlap of conserved essential genes between *Mtb* and *Mab.* **(B)** Enriched COG pathways of shared essential genes between *Mtb* and *Mab*. Pathways demarcated with an “*” indicate p-adj < 0.05.

**Supplemental Figure 3: Summary of gene essentiality category across *Mab* clinical isolates.**

**Supplemental Figure 4: Analysis of shared essential genes in *Mab* clinical isolates. Analysis of clade-specific essentials in *Mab* clinical isolates**. The HMM calls of the clade-specific genes across all 21 *Mab* isolates (ignoring genes with low-confidence calls, marked with an asterisk). The phylogeny of the 21 isolates is depicted above with the *massiliense* clade (includes all *massiliense* isolates) in yellow, the *abscessus* clade 1 in blue, and *abscessus* clade 2 in green. The highlighted gray genes are seen in Figure 5, depicting lineage-specific gene essentiality.

**Supplemental Figure 5: Pairwise Resampling Heatmap Matrix.** Each number in comparison represents the differentially required genes between two clinical isolates. Top left corner represents subsp. *massiliense – massiliense* comparisons. Bottom right comparisons represent subsp. *abscessus – abscessus* comparisons with strains more closely related to the ATCC 19977 reference strain.

**Supplemental Figure 6: Fold Repression of *pckA* and *sdhB.* (A).** Fold repression of *pckA* as measured by RT-qPCR. “*” signifies p < 0.05 by student’s t-test. **(B)** Fold repression of *sdhB* as measured by RT-qPCR.
